# Reduced-Risk Management of *Rhagoletis cerasi* Flies (Host Race *Prunus*) in Combination with a Preliminary Phenological Model

**DOI:** 10.1673/031.006.3401

**Published:** 2006-10-27

**Authors:** O. B. Kovanci, B. Kovanci

**Affiliations:** Uludag University, Faculty of Agriculture, Department of Plant Protection, Gorukle 16059 Bursa TURKEY

**Keywords:** Cherry fruit fly, degree-day, elevation, Tephritidae, yellow sticky traps

## Abstract

Seasonal flight activity of *Rhagoletis cerasi* (L.) (Diptera: Tephritidae) adults was monitored using yellow sticky traps at sweet cherry orchards under different management regimes in Bursa, northwestern Turkey, during 1997–1998. In the reduced-risk backyard orchards, soil ploughing in the fall or spring to destroy the pupae was combined with a single application of an insecticide, while conventionally managed orchards received six to seven insecticide applications for controlling adults. Traps in commercial orchards caught significantly fewer adults than those in reduced-risk backyard orchards. Levels of cherry fruit fly fruit damage were very low (0.1%) in commercial orchards, whereas infestation rates averaged 2.2% in reduced-risk orchards. A preliminary phenology model was developed for optimal timing of insecticide applications based on air temperature summations since 1 February. In the reduced-risk backyard orchards, the first flies were captured between 25 May and 2 June, corresponding to an average degree-day (DD) accumulation of 582.50 ± 10.50 DD at an altitude of 150 m. However, first adult emergence at 1170 m was recorded between 6 and 14 June, averaging 667.50 ± 14.50 DD. Adult emergence exhibited bimodal peaks in a single flight at low altitude but there was a single peak at high altitude sites. Total adult flight period averaged 459 ± 29.50 and 649 ± 25.50 DD at low and high altitude sites, respectively. Our prediction model suggests that the optimum spray-window for a single insecticide application occurs between 577.70 and 639.40 DD at 150 m and between 780.90 and 848.60 DD at 1170 m.

## Introduction

The true fruit fly genus *Rhagoletis* (Diptera: Tephritidae) consists of 65 described species distributed in the Holarctic and Neotropical regions, 17 of which were listed as important agricultural pests by White and Elson-Harris (1992). Of these, the European cherry fruit fly *R. cerasi* (L.) is the most serious pest of sweet cherries *Prunus avium* (L.) throughout Europe and in parts of temperate Asia (Fischer-Colbrie and Busch-Petersen 1989). *R. cerasi* has two host races, one feeding on *Prunus* spp. and the other on *Lonicera* spp. (Boller and Bush 1974). Adult females of the *Prunus* host race cause some damage by laying their eggs in the ripening sweet and sour cherries, but the major damage is caused by larval feeding in the fruit pulp. If left uncontrolled, the percentage of damaged fruits on medium to late ripening sweet cherry varieties can reach up to 100% (Fimiani 1984; Kovanci and Kovanci 2000).

Yellow sticky traps have proven to be useful tools for monitoring *R. cerasi* adults (Russ et al. 1973; Remund and Boller 1975). Monitoring the time and intensity of adult emergence with traps is critical for the better utilization of chemical control in the integrated pest management programs. A tolerance limit of 0–4% infestation of this pest for export has forced growers into intensive control programs to prevent infestations (Fimiani 1989). However, the restriction of pesticide use in agriculture by European Economic Community (EEC) regulation n. 2078/92 within the framework of IOBC/WPRS guidelines, coupled with the demand from export market for crops grown with less pesticide, has increased the interest for reduced-risk management of *R. cerasi.* It is possible to reduce the number of insecticide applications and to avoid improperly timed sprays via trapping but effective monitoring requires accurate timing of initial trap placement. In addition, trap catches, if employed as the sole indicator, have proven unreliable for determining the first emergence of *Rhagoletis indifferens* Curran flies in commercial orchards because of low populations (Song et al., 2003).

A complementary tool to trapping is the use of degree-day (DD) accumulations for predicting adult emergence based on the relationship between post-diapause pupal development and temperature. DDs are heat units that are accumulated above a specified base temperature during a 24-hour period (Baskerville and Emin, 1969). Using soil temperatures, a number of DD models have been developed for *R. cerasi* emergence in different parts of Europe, including Poland, Switzerland and Austria (Leski 1963; Boller 1964; Muller 1970). Leski (1963) reported that adult emergence began when pupae had accumulated 320 DD above a threshold of 7 °C starting from 1 January. The temperature sum of 430 DD above 5 °C was suggested by Boller (1964) but laboratory studies showed that 5 °C is too low as a base temperature (Baker and Miller 1978). However, Boller and Bush (1974) doubted the reliability of these predictions after they observed differences in emergence of flies, when kept at 25 °C following pupal storage at 2°C for 180 days, from different parts of Europe. Later, field studies carried out by Ranner (1988) indicated significant differences in emergence times and periods between flies of the *Prunus* and *Lonicera* race. Validation efforts using present models have not proven satisfactory when applied to local populations of *P. avium* host race of *R. cerasi* in Turkey (Kovanci 1998).

In addition, growers find it impractical to use soil temperatures for determining adult emergence. Although Aliniazee (1976) showed that both soil and air temperatures can be used for determining the emergence of *R. indifferens* flies based on the same lower developmental threshold of 5 °C, no such attempt was made for predicting the emergence of *R. cerasi* flies based on air temperature accumulations.

The major objectives of this study were to develop a reduced-risk management program and to predict the first emergence of *R. cerasi* flies for optimal timing of insecticide applications using DD accumulations as a complementary tool to trapping.

## Materials and Methods

### Description of the study sites

Experiments were conducted in small-scale backyard and large-scale commercial cherry sweet orchards at two locations, Bursa plain and Mount Uludag, in northwestern Turkey during 1997–1998.

The study sites were: 1) Doburca backyard orchard, central Bursa, located at an altitude of 150 m in the Bursa plain (40.20° N, 29.01° E), 2) Cukurca commercial orchard, east of Bursa, located at an altitude of 105 m in the Bursa plain (40.23° N, 29.03° E), 3) Sogukpinar backyard orchard located at an altitude of 1170 m in Mount Uludag (40.06° N, 29.13° E), 4) Sogukpinar commercial orchard located at an altitude of 1175 m in Mount Uludag (40.05° N, 29.14° E). Each backyard orchard was a 0.5 ha block of medium ripening ‘Bing’ and late-ripening ‘0900 Ziraat’ varieties while each commercial orchard was approximately 3 ha in size, consisting of late-ripening ‘0900 Ziraat’ sweet cherry and ‘Kutahya’ sour cherry varieties.

In backyard orchards, *R. cerasi* populations were managed under reduced-risk program. In this program, soil ploughing in the fall or spring to destroy the pupae was combined with a single application of diazinon (Hezudin 20 EM, Hektas, www.hektas.com) at 1.4 l in 1000 l of water per ha in 1997 and azadirachtin, derived from the seeds of neem tree (*Azadirachta indica* A. Juss), (Neem Azal-T/S 1% Azadirachtin, Trifolio-M GmbH, www.trifolio-m.de) at 31 in 10001 of water per ha in 1998 using a knapsack sprayer. Diazinon and azadirachtin application was made on 7 June and 30 May in Doburca backyard orchard in 1997 and 1998, respectively. Sogukpinar backyard orchard received one diazinon application on 25 June in 1997 and one azadirachtin application on 20 June in 1998.

Each commercial orchard received six to seven applications of malathion (Hekthion 65 EM, Hektas) at 1 l in 1000 l of water per ha using an airblast sprayer. Insecticide treatments began on 15 May and 1 June in Cukurca and Sogukpinar commercial orchards, respectively, in both years and continued on a weekly schedule until 5–7 days before harvest.

### Monitoring

Rebell type yellow sticky traps (Swiss Federal Research Station, Wadenswil, Switzerland) consisting of two crossed panels, coated with a layer of Tangletrap adhesive (The Tanglefoot Company, www.tanglefoot.com) were used to monitor the flight activity of adults. At each orchard, three traps were deployed in the exterior and southern side of tree canopy at 1.5–2 m height with a distance of 50 m from each other. Traps were placed in three different rows for each orchard in a diagonal pattern, one each in the northwestern and southeastern side of the orchard block, and one near the middle of the block. They were set up in early May. Traps were checked daily until the first sustained fly captures occurred and then adult abundance was monitored weekly until mid-August at each location.

### Fruit damage

Fruit damage was assessed for the presence of larva at harvest in late June at low altitude sites and early July at high altitude sites. Within each treatment, fruit damage was evaluated by picking 100 fruit arbitrarily from each of 5 trees per treatment and by inspecting each fruit and then cutting damaged fruit to check for internal infestation. All larvae were collected and identified to species.

### DD calculations

**Figure 1.**
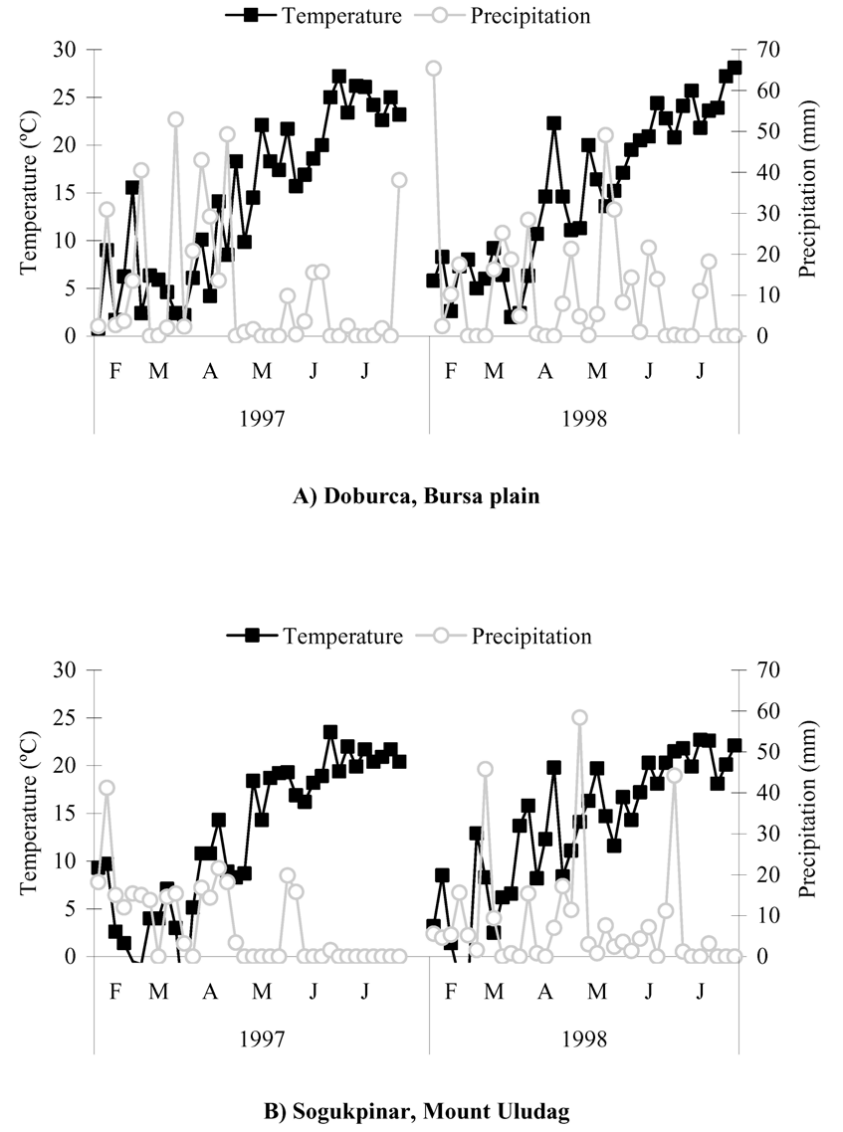
Five day average temperatures and total precipitation at a) Doburca and b) Sogukpinar during February-July in 1997 and 1998 (Bursa, Turkey).

Figure 1 shows the five day average temperatures and total precipitation in Doburca (Bursa plain) and Sogukpinar (Mt. Uludag) during the study period. The data were obtained from two national weather service stations in Bursa: (i) Hurriyet (100 m, 40.18°N 29.00°E) and (ii) Mount Uludag (1025 m, 40.10°N 29.20°E). Both stations are 1 km from the experimental areas. Based on the equation of Baskerville and Emin (1969), daily maximum and minimum air temperatures were used to calculate DDs above a developmental threshold of 7°C starting from 1 February (Leski 1963). The starting date of 1 February for the accumulation of DD was chosen because earlier experiments had shown that diapause termination in pupae occurred in the first week of February (Feron 1952).

Relative trap catches at each orchard were converted to percentage cumulative catch. Cumulative percentages were transformed to probits (Hrdy et al. 1996) and plotted against DD. Probit is a common transformation for linearizing sigmoid distributions of proportions (Armitage and Berry 1994). Probit transformation was performed to transform a typical sigmoid DD curve to a linear function. A linear regression, y = a + bx, was used to fit a relationship between fly catch and accumulated DD where y stands for probit transformation of the cumulative percentage of fly catches, and × stands for DD (SAS Institute 2001).

### Statistical analysis

This study was conducted using a randomized complete block design. The two treatments were the reduced-risk and conventional insecticide orchards. Both trap catch and fruit damage data were subjected to analysis of variance (ANOVA) and means were separated with Fisher's Protected LSD test (P < 0.05) (SAS Institute 2001). Fly catches in a total of 24 traps were used for statistical analysis and a total of 4000 fruits were analyzed for damage assessment. Mean cumulative trap catch values were used for analysis because of the many zeroes in the data. Trap catch and mean percentage fruit damage data were transformed using (log [*x* + 1]) and arcsine square root prior to ANOVA, respectively.

## Results

### Population dynamics of R. cerasi adults

**Figure 2.**
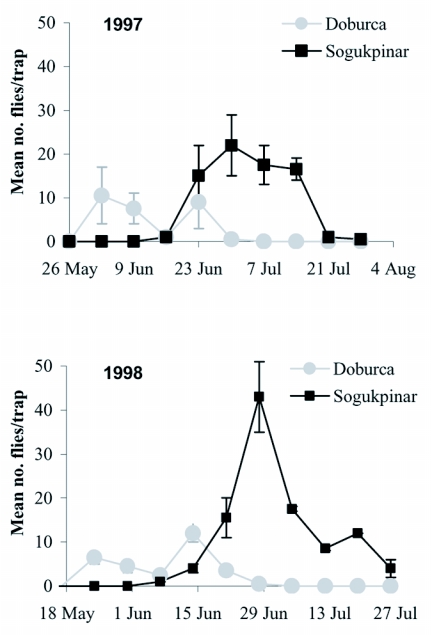
Mean weekly (±SEM) catches of Rhagoletis cerasi flies per yellow sticky trap in reduced-risk orchards at Doburca and Sogukpinar in 1997 and 1998 (Bursa, Turkey).

Mean weekly (±SEM) adult catches per yellow sticky trap for each reduced-risk backyard orchard at Doburca, the lowland site, and Sogukpinar, the mountain site, in 1997 and 1998 are shown in Figure 2. First fly captures occurred from 25 May (1998) to 2 June (1997) with an average trapping date of 29 May in Doburca. However, first adult emergence in Sogukpinar was recorded on 6 and 14 June in 1998 and 1997, respectively, with an average first detection time of 10 June. Hence, adults began emerging about 12 days later than those in Doburca.

Adult emergence in Doburca showed a bimodal pattern, with peaks of emergence centered around late May and mid-July whereas only one distinct peak was observed in Sogukpinar at the end of June. Traps failed to detect the first emergence of adults in intensively managed orchards because of low populations near or at zero, except for the Sogukpinar commercial orchard in 1998.

Mean cumulative fly catches per trap did not differ significantly between years (*F* = 2.39; df = 1, 13; *P* = 0.15). Sites located in Mount Uludag had significantly higher trap catches compared with those in the Bursa plain (*F* = 14.79; df = 1, 13; *P* < 0.01). Yellow sticky traps in commercial orchards caught significantly fewer *R. cerasi* adults than those in reduced-risk backyard orchards averaged over years (*F* = 71.65; df = 1, 13; *P* < 0.01). In 1997, the mean cumulative catch of flies was 51.00 ± 15.06 and 0.50 ± 0.50 adults per trap in reduced-risk and commercial orchards, respectively. Mean cumulative adult catch ± SEM increased to 67.50 ± 22.67 and 5.19 ± 2.59 adults per trap in reduced-risk and commercial orchards, respectively, in 1998.

### Fruit damage

Levels of cherry fruit fly fruit damage were very low (< 0.1%) in conventional insecticide orchards whereas infestation rates ranged from 1 to 3.4 % in reduced-risk orchards (Table 1). Although no significant differences were detected between the treatments when analyzed separately for each season (F = 11.93; df = 1,1; p = 0.20 for 1997, F = 23.36; df = 1,1; p = 0.15 for 1998), pooled ANOVA showed that there were significant differences between treatments averaged over years (F = 19.42; df = 1, 4; p = 0.01). Treatment effects were consistent over years and locations (F = 2.14; df = 1, 4; p = 0.21 for years, F = 4.61; df = 1, 4; p = 0.09 for locations).

**Table 1.**
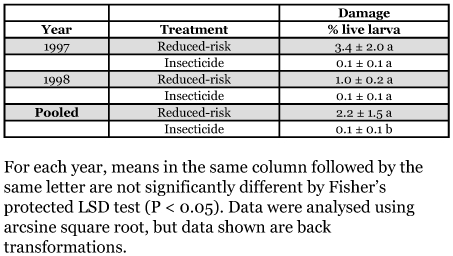
Mean (±SEM) percentage fruit damage averaged across two orchards under different management regimes (Bursa, Turkey)

### DD accumulations

DD accumulations since 1 February indicate that first adult emergence varied from 572 to 593 DD (582.50 ± 10.50) in Doburca and from 653 to 682 DD (667.50 ± 14.50) in Sogukpinar in 1997 and 1998, respectively. The mean accumulation for the beginning of adult emergence at the mountain site was 85 DD higher than those for Doburca. Apparently, the effect of the cooler mean temperatures at the mountain site was reflected in the timing of adult emergence.

Adult emergence exhibited bimodal peaks in a single flight at the Doburca orchard; the first peak occurred from 597 to 619 DD and the second peak occurred from 874 to 884 DD depending on years. In contrast, there was a single peak emergence of adults at the Sogukpinar orchard, with a peak from 890 to 946 DD. Adult flight ended at 1001 and 1081 DD in Doburca in 1997 and 1998, respectively. No adults were caught after 1276 and 1356 DD in Sogukpinar in same order. Thus, total adult flight period ranged from 429 to 488 DD in Doburca and from 623 to 674 DD in Sogukpinar.

The linear relationship between temperature accumulations and cumulative percentage trap catches of *R. cerasi* adults at Doburca and Sogukpinar backyard orchards in 1997 and 1998 are presented in Table 2. In all cases, linear correlations were statistically significant. Correlation coefficients were also high, ranging from 0.92 to 0.99. Comparison of the linear equations obtained from Doburca with Sogukpinar revealed that line slopes were not significantly different (F = 0.74; df = 1, 29; p = 0.40). Additionally, the 95% confidence intervals (CI) of the correlations were quite narrow, especially for the slopes. Unlike these high linear correlations between temperature and adult emergence, there was no clear relationship between adult emergence and precipitation.

**Figure 3.**
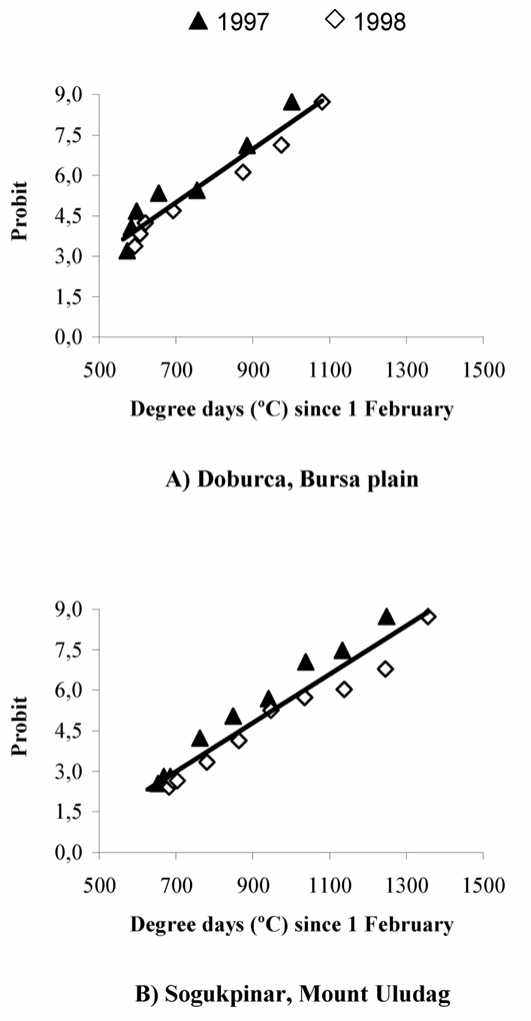
Relationship between probit transformation of the cumulative percentage of *Rhagoletis cerasi* flies caught in yellow sticky traps and degree-day accumulations above 7°C since 1 February in reduced-risk orchards at a) Doburca and b) Sogukpinar in 1997 and 1998 (Bursa, Turkey).

When DD accumulations for both years were combined separately for each location, linear model predictions gave a good fit to the actual trap catches (Figure 3). The model predictions for the mean DD accumulation values corresponding to 10, 25, 50 and 75% of actual trap catches are summarized in Table 3. The differences between the DD predictions and the observed values ranged from 1 to 2 days. For example, the accumulated DD values for 10% of trap catches in Doburca was predicted at 578 DD and actual mean 10% catch occurred at 572 DD when averaged across years, indicating a highly accurate predictive model. Similarly, the observed and predicted dates of 10% adult emergence at Sogukpinar were similar, 766 and 781 DD, respectively.

## Discussion

The results of this study clearly showed that there were significantly more flies captured in yellow sticky traps in the reduced-risk backyard orchards compared with the conventionally treated orchards. Similarly, levels of fruit damage in the reduced-risk orchards were significantly higher than those in the conventional insecticide orchards when data were pooled over years. It seems that one spray application may not provide adequate protection at the low altitude site (Doburca) because of uneven ripening of cherries and the consequent bimodal flight pattern. Likewise, an additional insecticide application may be necessary at the high altitude sites (Sogukpinar) due to prolonged emergence pattern of adults.

**Table 2.**
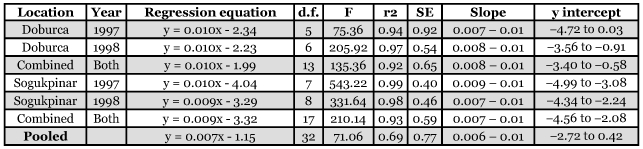
Relationship between probit transformation of the cumulative percentage of *Rhagoletis cerasi* flies caught in yellow sticky traps and degree-day accumulations above 7°C since 1 February in reduced-risk orchards at Doburca and Sogukpinar (Mt. Uludag) in 1997 and 1998 (Bursa, Turkey)

Based on our observations, yellow sticky traps were unreliable as an accurate forecasting tool for detecting the first adult emergence in intensively managed conventional orchards, where *R. cerasi* populations were very low. This is in agreement with the findings of Song et al. 2003 who reported that traps failed to determine the first emergence of *R. indifferens* adults in orchards where populations were low. Since the infestation tolerance limit is low (0–4%), commercial growers are even concerned about low populations, which can cause high level of infestations (Fimiani 1984).

As a complementary tool to trapping, we developed a DD model for accurate timing of insecticide applications. It is apparent that our DD model can predict the time of adult emergence with a high accuracy. Model predictions showed a good linear fit to the actual trap catches. The widest differences between the predicted and observed DDs for the various percentages of the adult emergence were only 36.34 and 41.92 DD (Table 3), which corresponds to a difference of 2 days based on the mean June temperatures of 19.7 and 22.4 at Sogukpinar (the mountain site) and Doburca, respectively. Differences between the regression line slopes of Doburca and Sogukpinar were not significant. This information suggests that the model can be generalized, however, the lower correlation coefficient of the pooled data from all locations, when compared with high correlation for each location separately, shows the need for incorporation of other biological factors into this model to represent the actual development process of local populations (Song et al. 2003).

**Table 3.**
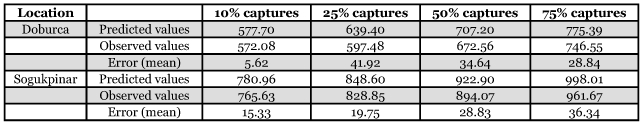
Predicted and observed values of degree-day accumulations above 7°C since 1 February corresponding to 10, 25, 50 and 75% of *Rhagoletis cerasi* adult captures in yellow sticky traps deployed in reduced-risk orchards at Doburca and Sogukpinar, Mt. Uludag (Bursa, Turkey)

In average, *R. cerasi* adults emerged 12 days earlier in Doburca than those in Sogukpinar but the difference in DD accumulations required for emergence was only 85 DD. Varying temperature sum requirements for adult emergence between locations may be explained either by the differences in timing of diapause termination or by the differences in post-diapause development (Baker and Miller 1978) likely due to cooler temperatures in Sogukpinar. Further experiments are necessary to confirm these hypotheses.

Adult populations in Sogukpinar not only emerged later but also showed a prolonged emergence pattern compared with those in Doburca. In laboratory studies, Boiler and Bush (1974) also found late and prolonged emergence curves for pupae from three out of 17 sites which they were collected. They suggested that these emergence curves may be associated with the uneven ripening of cherries in these areas. However, prolonged emergence was not found to be the case in Doburca, where medium and late-ripening varieties were mixed with each other. One possible explanation for the prolonged emergence pattern at the Sogukpinar orchard may be the presence of an alternative host, sour cherries, which bears fruit later in the season than sweet cherry. Sogukpinar backyard orchard was surrounded by many sour cherry orchards and sour cherry infestations can reach up to 33% in this area (Kovanci 1998). Evidently, the ripening period of sweet cherries regulates the mean emergence time. However, sour cherries provide an alternative oviposition site and their ripening period can determine the range of the emergence time. This may be a bet-hedging or risk spreading strategy which may give a selective advantage because the offspring of any given female emerge over an extended time period, as insurance against poor conditions in some years (Hopper 1999). In a heterogeneous environment, larval oligophagy together with the ability of local populations to adapt to the chemical quality and ripening period of the dominant host plant also makes the opportunistic use of low quality host species, in this case sour cherries, possible (Tikkanen 2000). This phenological adaptation phenomonen was also reported in *Operophtera brumata* L. populations (Tikkanen 2002).

Based on adult captures on yellow sticky traps, *R. cerasi* adults exhibits a bimodal emergence pattern in a single flight at the Doburca orchard, which consisted of medium ripening ‘Bing’ and late-ripening ‘0900 Ziraat’ varieties. Likewise, bimodal peaks in apple maggot *Rhagoletis pomonella* (Walsh) adults were observed in western North Carolina, USA (J.F. Walgenbach, personal communication). Potential reasons for this bimodal activity pattern in *R. cerasi* adults are unknown, but may be attributed to sweet cherry cultivar on which larvae feed, variation in diapause requirements, and/or a genetically linked trait. Whether sweet cherry cultivar affects the developmental rate of *R. cerasi* larvae, and thus the time when they enter and complete diapause development is unknown.

Our field observations indicated that 10% emergence of adults coincided with the ripening period of sweet cherries when the fruits start turning from a bright green to a straw-yellow. It is common for backyard cherry growers to make one insecticide application during this period and usually no additional spray is applied because of approaching harvest time and economic constraints. However, insecticide applications for control of *R. cerasi* adults can be best timed by using the mean DD values corresponding to the various percentages of the adult emergence. Our prediction model suggests that the optimum spray-window for a single insecticide application occurs between 577.70 and 639.40 DD in Doburca and between 780.90 and 848.60 DD in Sogukpinar. This observation has not yet been tested and the efficacy of control should be evaluated for the various percentages of the adult emergence. Yet, our DD model can help pest managers to convince commercial growers to stop using calendar-based sprays. Optimal timing of insecticide sprays could reduce the overall number of applications during the growing season and thus reduce control costs and the risk of non-target effects and environmental contamination. In conclusion, our results suggest that the use of DDs in combination with traps would provide a safer and more cost-effective management system for *R. cerasi* populations.
